# Monthly Summary

**Published:** 1897-05

**Authors:** 


### Monthly Summary.

AN EMPTY PULP CANAL.—By Dr. Hyatt, Brooklyn, N. Y.—I came across a case last summer which was new to me. I was cutting down a bicuspid tooth to crown it. There was a cavity—not a large one—on one side, and in removing the decay I found an exposure of the pulp. I treated it, capping it with asbestos paper and inserting cement filling probationally. The patient then left town, returning in a week, when I proceeded to complete my operation. In grinding the tooth I removed the cement filling and the asbestes, but to my amazement I found the pulp chamber empty wholly to the end of the root. When I first exposed that pulp, it bled freely, yet within a week's time there was nothing there; the canal was perfectly dry. The patient, who was an old friend of mine, said that probably it was like some dead trees, which we have all seen, on one end of which there is a little green twig. In this case the pulp was probably dead before I commenced operating on it, and perhaps just the corner which I touched had a bit of life.—*Items of Interest.*

---

UNNECESSARY SACRIFICE OF DENTAL PULPS.—By Dr. W. A. Siddall.—There is nothing in dentistry which requires more skill and thought and good judgment than to properly place anchorage for a filling without danger to the pulp. To be able to tell where the pulp is in a tooth and to so place anchorage that the filling will not be injurious to the life of the pulp, requires the greatest judgment.

There are so many things to be considered—the age of the patient, the density of the tooth, any peculiar shape of the tooth or cavity. We must look out for any unusual location of the pulp or unusual length of the horns of the pulp.

I believe that there are a large number of pulps sacrificed unnecessarily by a lack of care and judgment in the preparation of cavities.

There is no question but that the use of hot air in connection with a certain medicament for sensitive dentine, a few years ago, resulted in the death of a great many pulps, and we should be careful to know what effect any new treatment for the same purpose may have on the pulp.

No doubt many pulps will be sacrificed by the use of cocaine and cataphoresis.

It is often a difficult thing to diagnose an exposed or nearly exposed pulp, and especially is this so when the dentine is insensible to pain. I would not underestimate the value of a method whereby the dentine can be desensitized, but would urge that unusual care be exercised in its use.

Whether cataphoresis will prove to be detrimental to the welfare of the pulp remains to be seen, but certainly unless great care is used many pulps will be destroyed by its use.—*Ohio Dental Journal.*

THE DESTINY OF THE THIRD MOLAR.—By D. L. Hertig, D. D. S.—I have never heard any satisfactory explanation as to why the wisdom teeth put in their appearance from ten to twelve years after an abundant supply of the organs of mastication has already been provided.

Not a dentist in the land will disagree with me when I say that 28 teeth are enough for any man, be he Zulu or professor; that is, if the aforesaid 28 can be kept in good condition, which in a great many cases is impossible.

Here is where the utility of the third molar manifests itself; here is where its late arrival shows as much forethought on the part of the Supreme Designer as does the early advent of the six year molar. Not because the lapse of ten or twelve years allows the jaws to develop so as to receive it. No!

Had he willed it so, the Ordainer of all could have caused the same development to have taken place in the youth of 14, for the reception of the third as did take place in the child for the reception of the first and second molars.

I think a different plan was intended.

I regard the third molars, coming as they do years after a full complement of teeth has been supplied, as auxiliaries, reinforcements in fact to the original set for the accidental loss of one or more of them. We see this illustrated time and again. We cannot fail to notice the important duty performed by these organs.

I have observed many cases where the timely arrival of the wisdom tooth has filled the space and supplied the loss of a valuable grinder. In this way it fulfills a more important mission than if it came merely as an addition to an unbroken arch.—*Ohio Dental Journal.*

---

DRAWING ROOM:—"Do you know," said the man who was going to have a tooth pulled, "I don't think 'dental parlor' is a good phrase."

"No?"

"Drawing room would be much better."—*Woman's Journal.*

